# Distributed Patterns of Event-Related Potentials Predict Subsequent Ratings of Abstract Stimulus Attributes

**DOI:** 10.1371/journal.pone.0109070

**Published:** 2014-10-01

**Authors:** Stefan Bode, Daniel Bennett, Jutta Stahl, Carsten Murawski

**Affiliations:** 1 Melbourne School of Psychological Sciences, The University of Melbourne, Parkville, Victoria, Australia; 2 Department of Finance, The University of Melbourne, Australia, The University of Melbourne, Parkville, Victoria, Australia; 3 Department of Psychology, University of Cologne, Cologne, Germany; Max Planck Institute for Human Cognitive and Brain Sciences, Germany

## Abstract

Exposure to pleasant and rewarding visual stimuli can bias people's choices towards either immediate or delayed gratification. We hypothesised that this phenomenon might be based on carry-over effects from a fast, unconscious assessment of the abstract ‘time reference’ of a stimuli, i.e. how the stimulus relates to one's personal understanding and connotation of time. Here we investigated whether participants' post-experiment ratings of task-irrelevant, positive background visual stimuli for the dimensions ‘arousal’ (used as a control condition) and ‘time reference’ were related to differences in single-channel event-related potentials (ERPs) and whether they could be predicted from spatio-temporal patterns of ERPs. Participants performed a demanding foreground choice-reaction task while on each trial one task-irrelevant image (depicting objects, people and scenes) was presented in the background. Conventional ERP analyses as well as multivariate support vector regression (SVR) analyses were conducted to predict participants' subsequent ratings. We found that only SVR allowed both ‘arousal’ and ‘time reference’ ratings to be predicted during the first 200 ms post-stimulus. This demonstrates an early, automatic semantic stimulus analysis, which might be related to the high relevance of ‘time reference’ to everyday decision-making and preference formation.

## Introduction

What information is automatically extracted during exposure to stimuli in our environment? This question is important considering that humans can, seemingly effortlessly, make fast decisions based on sometimes even very abstract stimulus information. For example, one fundamental type of everyday decision involves choosing between rewards that are available right now, satisfying an immediate desire, and alternative, subjectively equally valuable rewards that only become available in the future, requiring the decision-maker to be patient. For example, a delicious meal in a fancy restaurant and a wedding party might both be equally rewarding to the decision-maker in today's terms, but they might be available at different points in time – the former immediately, and the latter only in a few months or even years. It has been shown that the mere exposure to rewarding stimuli can prime people to change their choice behaviour in favour of either an immediate reward or a delayed reward in delay discounting tasks in which participants make intertemporal choices between smaller-but-sooner and larger-but-later monetary rewards [1]–[3]. Interestingly, this is true even for priming stimuli that are seemingly unrelated to the decisions. Priming towards immediate gratification in delay discounting tasks has been demonstrated using erotic images, food items, status symbols (such as cars), computer brand logos and fast food logos as primes [4]–[8]. Priming towards delayed gratification, on the other hand, has been shown by means of creating vivid mental images of positive future life events [9], [10].

One explanation for the effect of rewarding stimuli on subsequent intertemporal decisions is that decision-makers might have fast access to the *semantic attributes* of stimuli by means of a rapid, automatic semantic analysis during perceptual processing. Such a semantic analysis would also involve extracting the stimuli's reference to time, i.e. whether they are subjectively more strongly related to the present or to the future (henceforth termed *time reference*). For example, a delicious food item might activate present-related thinking, while an elderly happy couple at a lake might be subjectively related to the concept of the future. Thus, a precursor to understanding the described priming effects is to assess first whether an immediate semantic analysis takes place during perceptual processing of stimuli. Such an analysis might not even require awareness of the relevance of different semantic dimensions.

We reasoned that, if an automatic semantic analysis of the *time reference* takes place, brain activity immediately following stimulus exposure will contain information about the outcome of this cognitive process. Recent studies have successfully used functional magnetic resonance imaging (fMRI) to investigate brain activity associated with, or predictive of, automatic processing of stimulus attributes. For example, brain activity has been shown to be associated with attractiveness ratings for faces even though the experimental task was to judge gender [11]. It has further been shown that brain activity reflected processing of unattended stimuli on a category level [12], as well as personal preferences for complex semantic stimuli when attention was withdrawn from preference-relevant dimensions [13], [14]. One shortcoming of fMRI, however, is its rather poor temporal resolution, which does not allow for the investigation of the time course of fast information processes of an abstract semantic dimension on a millisecond time scale. Importantly, no study to date has investigated the processing of the abstract semantic dimension of *time reference*, which has been shown to be highly relevant for everyday temporal decision making. In the present study, we aimed to examine whether neural activity patterns point towards semantic analysis of stimuli already shortly after the initial perceptual processing within the first few hundreds of milliseconds, and whether this analysis can take place without explicit attention to specific stimulus dimensions. Such a finding would also provide a foundation and theoretical basis for the explanation and further investigation of time-related decision biases in intertemporal choice.

In order to address this question, the present study used electroencephalography (EEG). Specifically, we investigated whether the subjective evaluation of the abstract dimension *time reference* of rewarding images was predictable from brain activity, measured with high temporal resolution during the first several hundred milliseconds following stimulus onset. We presented our participants with invariably positive-valence images in the background, taken from different categories (food, money, status symbols, happy aging, family, romance, erotic) as recent studies have shown that these stimuli influence intertemporal choice [4], [5], [7]–[10]. To render the images task-irrelevant, as it was the case for most choice priming studies, participants were asked to perform an attention-demanding foreground task while ignoring the images in the background [13]. This manipulation further had the effect of directing attention away from the stimuli while still allowing for their full perceptual encoding [15]. Following the experiment, participants were asked to rate a) how arousing/exciting they found each stimulus to be (*arousal*), and b) the extent to which each stimulus was present-related or future-related (*time reference*). *Arousal* was included as a control dimension as it constitutes a fundamental, more concrete stimulus dimension that, unlike *time reference*, is likely to involve additional emotional and physiological responses, thus making it plausible that different arousal levels would be reflected in differences in brain activity [16], [17] in an early processing period. Furthermore, this allowed us to control for the possibility that prediction of the *time reference* of the images was confounded by differences in *arousal*. Importantly, participants were not informed before the EEG experiment that they would be asked to rate the stimuli later on, nor were they aware of the relevance of these particular stimulus dimensions.

We investigated whether participants' subjective post-experiment ratings were predictable from early components of the event-related potential (ERP), including the N1, the N2, the early posterior negativity (EPN) and the P3, which can typically be observed during the first few hundred milliseconds following stimulus onset, and might also be related to the processing of abstract stimulus features. These components have been discussed to reflect a variety of aspects of visual stimulus processing which were probably involved in our task, including the early allocation of attention (N1 [18]–[20]), semantic stimulus categorisation (N2, P3 [21]), emotional evaluation and attention (N1, N2, P3 [22], [23]), establishing internal stimulus representations (P3 [24]), duration of evaluation of stimuli (P3 latency, for review see [25]), differences in valence and arousal (N2, P3 [17]), context updating/closure (P3 [26], [27]), arousal levels (EPN, P3 [28], [29], for review on the P3 see [30]), and perceptual decision-making (P3 [31]).

However, taking into account that our stimuli were semantically rather complex, we anticipated the involvement of multiple brain regions in the semantic *time reference* analysis, which, in turn, might lead to a more diffuse distribution of signals, diminishing clear differences at single electrodes. To account for the possibility that the entire pattern of brain activity rather than specific components measured at single channels predicted the ratings, we used highly sensitive multivariate pattern analyses for ERPs [32]–[37]. As we wanted to test whether rather subtle information about *time reference* is processed in an early time period (which would explain the above-mentioned priming effects), this more sensitive technique might be better suited to investigate the time course of information processing compared to traditional ERP techniques. A previous study showed that physical stimulus features can be extracted from EEG signals while attention to the stimulus was systematically manipulated [38]. Here, we extended this approach and investigated whether abstract cognitive dimensions of stimuli can be predicted from patterns of EEG signals while attention was withdrawn from the stimuli. Specifically, a novel support vector regression analysis (SVR) approach for EEG was used to predict the ratings from distributed spatio-temporal patterns of ERPs for a series of consecutive time windows after stimulus onset. We hypothesised that if a fast and automatic evaluation of the *time reference* dimension takes place during the early stages of visual processing, this should be reflected in predictive differences between patterns of ERPs, reliably related to the post-experimental ratings.

## Materials and Methods

### Participants

Twenty right-handed participants, mostly students of the University of Melbourne, Australia, with normal or corrected-to-normal visual acuity, gave written informed consent to participate in the study and were compensated with AUD 20 for their time. Four data sets had to be discarded due to excessive blinking and muscle artefacts, and for another two data sets, one rating dimension each (*time reference* for one participant, *arousal* for the other one) had to be excluded because participants made use of only extreme ratings, eliminating the required variability in the data. The final sample consisted of 16 participants (10 female, mean age  = 22.2 years, range 18–31).

### Ethics statement

The experiment was approved by the ethics committee of the University of Melbourne (No. 1033349.3) and was conducted according to the Declaration of Helsinki.

### Stimuli and procedures

Stimuli were positive-valence images from different categories from the International Affective Picture System (IAPS [39]). All images were chosen such that they were only positive on the *valence* dimension, with norm-ratings>5 (from 1 =  low to 9 =  high; *M* = 6.98, *SE*  = 0.12; range 5.22–7.77) (for norms see [40]). This was done in order to eliminate strong differences in preference between stimuli, as well as to rule out automatic negative emotional reactions. Images were preselected based on ratings by an independent sample of 57 participants (39 female, mean age  = 21.6 years, range 18–34) on the *time reference* dimension using a scale from 1 =  “strongly associated with the present” to 9 =  “strongly associated with the future”. Twenty-four images (of originally 46) that optimally covered the entire scale were selected for the EEG experiment (for IAPS image codes see Table 1), which was conducted using a different sample of participants (see above), who had not been exposed to the stimuli before. Each image was 512×384 pixels in size (11.5×8.5 cm on the screen). During the EEG experiment, participants were seated with their chins resting comfortably on a chin rest at 50 cm distance from the screen, such that stimuli subtended 13.12°×9.72° visual angle.

**Table 1:** Image Ratings.

			Rating					
Image			Arousal(1–9)^a^		Time ref.(1–9)^b^			
#	IAPS Code	IAPS Description	*M*	*SE*	*M*	*SE*	*r*	*P*
1	4599	Romance	6.81	.50	4.88	.62	−.21	.44
2	4650	Erotic Couple	5.75	.56	6.25	.48	−.19	.48
3	4676	Erotic Couple	6.94	.45	6.69	.57	−.19	.49
4	4693	Erotic Couple	6.63	.51	6.38	.61	−.01	.97
5	4695	Erotic Couple	6.75	.46	6.38	.69	−.13	.62
6	4645	Erotic Couple	6.38	.48	5.69	.70	−.05	.86
7	7405	Cupcakes	6.19	.64	3.06	.49	.35	.19
8	7470	Pancakes	6.19	.60	2.13	.32	.12	.67
9	7400	Candy	5.06	.51	3.56	.47	.12	.65
10	7480	Pasta	6.81	.49	3.69	.60	.07	.80
11	7351	Pizza	5.94	.58	3.44	.57	.04	.88
12	7481	Food	5.56	.51	4.81	.66	−.56	.02*
13	2550	Couple	5.00	.68	8.31	.30	.27	.31
14	2370	Three Men	3.56	.57	7.56	.39	.23	.39
15	2510	Elderly Woman	5.00	.74	7.88	.52	.00	1
16	2495	Man	2.62	.59	6.38	.60	−.03	.91
17	2500	Man	4.13	.79	7.75	.48	−.09	.75
18	4626	Wedding	7.06	.48	8.13	.33	.32	.23
19	8531	Sport Car	6.63	.51	7.31	.41	.20	.46
20	7530	House	6.06	.62	7.88	.34	−.03	.90
21	8502	Money	7.50	.33	6.25	.61	−.10	.72
22	8500	Gold	7.00	.49	6.63	.51	.15	.56
23	2152	Mother	5.81	.54	6.88	.48	.23	.38
24	2165	Father	6.19	.56	7.63	.39	.48	.06

Note: Only participants' ratings that were included in the respective EEG analyses are included here (*N* = 15); *M* =  mean; *SE*  =  Standard error; *r* are Pearson-correlation coefficients for the correlation between *arousal* ratings and *time reference* ratings across participants; ^a^ higher ratings indicate more arousing images; ^b^ higher ratings indicate future-related images, lower ratings indicate present-related images; IAPS Code and Description refer to the original labels in the International Affective Picture System; *P<.05 (uncorrected).

**Figure 1:**
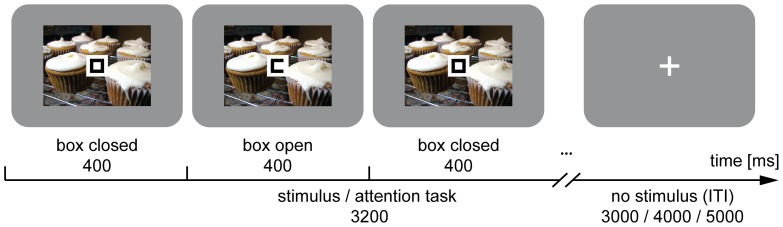
Paradigm. On each trial, one of 24 IAPS images was presented in the background for 3.2 s while participants engaged in an attention-demanding foreground task. They continuously monitored the random opening of a box (alternating between 400 ms closed, 400 ms opened) and responded to the side of each opening with a left or right button press using the left and right index finger. This task phase was followed by a jittered delay of 3 s (25%), 4 s (50%) or 5 s (25%) in which only a white fixation cross was shown. Each image was shown 3 times per block (total of 72 trials per block), and participants finished 6 blocks (total of 432 trials) with an individually randomised order of images for each participant and each block.

On each trial (Figure 1) in the EEG study, one image was presented in the background for 3.2 s while participants engaged in an attention-demanding foreground task. Participants' only task was to monitor a box, which opened randomly to the left or right (alternating between periods of 400 ms closed and 400 ms open, always starting with a closed box; 0.5×0.5 cm; visual angle 0.57°) overlaid on the centre of the image, and to respond immediately to the side of each opening with a left or right button press on a response box, using the left and right index finger respectively. Due to its pace, the task was highly demanding, as has been demonstrated before [13], [15]. As in previous studies, the task was used to focus attention away from the images, allowing for automatic stimulus processing rather than conscious reflection on their meaning. Engagement with the task did not prevent stimulus processing in general, but it was intended to prevent uneven allocation of attentional resources to different stimuli. The image presentation/task phase was followed by a jittered delay of 3, 4, or 5 s, during which a white fixation cross was shown. Each image was presented 3 times per block (total of 72 trials per block) in six blocks (total of 432 trials) with an individually randomised order of images for each participant and each block.

After the EEG experiment, participants performed a computer-based rating task on the same images that were shown in the EEG part, presented twice in a new individually randomised order. Participants were asked to rate each image on a nine-point scale, once with respect to how arousing the image was and another time with respect to the *time reference* dimension, as described above. The exact ratings were converted off-line into rating increments (1–3 “low arousal”/“present”; 4–5 “medium arousal”/“intermediate time”; 6–9 “high arousal”/“future”) in order to have a roughly equal numbers of rating increments for the following analyses. Participants were not aware of the subsequent rating task when participating in the EEG experiment, and they indicated not having been aware of the importance of the stimuli's *time reference* and *arousal* dimensions when asked.

### Low-level image features

To control for the effect of immanent low-level features of the images on the ratings, eleven low-level image feature parameters were determined separately for each of the 24 images: three values of the RGB colour space (R, G, B), three RGB value ratios (R/G, R/B, G/B), the mean RGB colour value, the mean, median and standard deviation of the images' luminance, as well as the mean spatial frequency. Mean spatial frequency was extracted using a Fourier transformation on each grey scaled image. The set of low-level image features was chosen based on previous studies using these features insofar as they were applicable to our type of stimuli [41], [42]. Correlations were used to assess relationships between these values and mean rating scores (i.e. rating scores averaged across all participants), as well as with the individual rating scores (averaged across all participants after Fisher-Z-Transformation of individual correlations), for *arousal* and *time reference* separately. Furthermore, multiple regression analyses were used to predict the post-experiment ratings for *arousal* and *time reference* separately from the combination of all low-level image feature values.

### EEG data recording, pre-processing and analysis

The electroencephalogram (EEG) was recorded from 64 scalp electrodes (Fp1, Fpz, Fp2, AF7, AF3, AFz, AF4, AF8, F7, F5, F3, F1, Fz, F2, F4, F6, F8, FT7, FC5, FC3, FC1, FCz, FC2, FC4, FC6, FT8, T7, C5, C3, C1, Cz, C2, C4, C6, T8, TP7, CP5, CP3, CP1, CPz, CP2, CP4, CP6, TP8, P9, P7, P5, P3, P1, Pz, P2, P4, P6, P8, P10, PO7, PO3, POz, PO4, PO8, O1, Oz, O2, Iz). The active Ag/AgCl electrodes (interfacing a BioSemi Active Two system, using ActiView acquisition software) used an implicit reference during recording and were re-referenced offline against the average of both mastoids. The vertical and horizontal electrooculogram (vEOG/hEOG) were recorded from electrodes infraorbital and next to the left eye. The EEG was continuously recorded at a sampling rate of 512 Hz, applying an online 0.1 to 70 Hz band-pass filter. The EEG was analysed time-locked to the onset of picture presentation using a baseline period of 100 ms before and 700 ms after the stimulus onset (covering the most important components of the event-related potentials). A standard 50 Hz notch filter was applied to remove electrical noise from the data. All data were first screened for technical artefacts (±500 µV). The cleaned data set was then subjected to an independent components analysis (ICA) as implemented in the EEGlab-Toolbox [43] to identify and remove components related to eye movements and eye-blink artefacts. Subsequently, an additional, stricter artefact screening was performed in which contaminated trials with max/min amplitudes exceeding ±200 µV were rejected. A standard current source density (CSD) analysis of the ERP was performed for each of the 64 electrode sites. The CSD signals were computed for each electrode site by taking the second derivative of the distribution of the voltage over the scalp and accounted for the curvature of the head using a spline algorithm [44]–[46]. CSD analysis has the advantage of achieving higher independence of the location of the reference electrode from other electrodes and higher topographical accuracy of the CSD signals [47]. Note that CSD analysis is a standard procedure in the ERP literature (e.g., for N1 and P3 see [48], [49]; for review see [50]).

Average CSD-ERP waveforms were computed separately for each participant and for each rating increment (*arousal*: high/medium/low; *time reference*: present/intermediate/future). A 30-Hz low-pass filter was applied for the analysis of the following components of the event-related potentials involved in early stimulus processing. The selection of electrodes for the ERP analyses was based on the local maximum of each component and corresponded to the typical electrode sides described in the literature: CSD-N1 at FCz (determined as the most negative amplitude in the range from 50 ms to 150 ms separately for each condition and each participant), CSD-N2 at Pz (from 150 ms to 300 ms) and CSD-P3 at Pz (the most positive amplitude in the range from 300 ms to 700 ms [30]). The early posterior negativity (EPN) was calculated as the mean voltage within the interval from 280 to 320 milliseconds following stimulus onset, collapsed separately across left and right occipital and parieto-occipital electrodes O1, P9, PO7 and O2, PO8, P10 [29]. All components were analysed with repeated-measures ANOVA including within-subjects factors of rating increment condition (low/medium/high *arousal*, or present/intermediate/future *time reference*, as relevant) and laterality (left, right). Greenhouse-Geisser correction was applied for violations of the assumption of sphericity where relevant.

Additionally, a linear support-vector regression (SVR) analysis was performed on the data without applying the 30-Hz low-pass filter. For this, each participant's trials were again sorted into rating increments, separately for both post-experiment rating items. A balanced number of exemplars (trials) for each participant were randomly selected for each rating increment and each condition, in order to avoid sample-size biases. The classifier then analysed the first 400 ms after onset of stimulus presentation (i.e. before the first box opening event that required a motor response) in time steps of 40 ms, starting at stimulus onset, with an analysis time window which moved through the 400 ms post-stimulus data in steps of 20 ms (i.e., 0–40 ms, 20–60 ms, etc.). The data of all 64 channels within each window were transformed into vectors (each of the 20 data-points within this time window for each of the 64 channels served as individual features), representing the spatio-temporal patterns associated with the ratings. The SVR was performed using LIBSVM ([51]; parameter S = 3 and C = 0.1). The outcome of each multivariate regression analysis was a correlation of the predicted label/rating and the true label/rating, thereby quantifying how well the ratings could be regressed from distributed patterns of CSD-ERPs within a given time window. First, in order to estimate the regression function, data from all trials were randomly divided into 10 equally-sized bins. Of those, 90% were randomly drawn and used for training the model, and the remaining 10% of the data were used for testing. A ten-fold cross-validation procedure was applied for which each 10% proportion of the data was used for testing once while the other 90% of the data were used for training. Subsequently, to avoid drawing biases, the entire analysis was repeated ten times with newly drawn 10% data proportions, resulting in a total of 100 analyses. The correlation coefficients derived from each analysis were Fisher-Z-transformed, and results were averaged across all analyses. A significant correlation means that brain activity patterns were predictive for the subsequent ratings. The same analysis was conducted using randomly shuffled labels for each participant and each analysis time window in order to obtain an empirical distribution of regression results under the null hypothesis for each analysis step. Group level statistical analyses (*T*-tests using a threshold of *P<*.05) compared the empirical results with the shuffled labels SVR results for each time window, in order to test whether the respective activity patterns could be used to regress the true individual ratings for all stimuli.

Finally, we asked how well the ratings could be predictive from each channel separately during the time periods that allowed for significant multi-channel prediction. In order to achieve high comparability between rating conditions, we restricted our analysis to the series of consecutive time bins that showed the first significant peaks for both rating dimensions (*time reference*: combined bins 100 ms and 120 ms; combined bins 160 ms, 180 ms, and 200 ms; *arousal*: combined bins 180 ms and 200 ms). For each of these temporal decoding analyses, new pattern vectors were constructed for each channel separately, based on all temporal information (one data point per 2 ms) within these time periods. The same multivariate SVR was then performed on these vectors to regress the ratings, again separately for each rating dimension. As for the spatio-temporal analysis, data from all trials were again randomly divided into 10 equally-sized bins and 90% were randomly drawn and used for training the model, while the remaining 10% of the data were used for testing. This again resulted in a ten-fold cross-validation procedure. Note that this analysis cannot reveal the origin of predictive information from the multi-channel SVR; it was only used to illustrate how well ratings could be predicted from single channels in the respective time periods.

## Results

### Attentional foreground task

For the analysis of the foreground attention task, two participants' data had to be excluded because of technical problems during recording of button presses. Participants responded accurately to the box opening with *M* = 71.6% (*SE*  = 3.93) correct responses within the 400 ms time-window, demonstrating that they were highly engaged in the task, as instructed. Performance accuracy was highly similar for all background images (ranging from 67.4% to 76.2% in individual participants). The average response time across participants for the fixation task was 316 ms (SD  = 20 ms; range 291–355 ms). Importantly, none of the individual post-hoc ratings were significantly correlated with the accuracy in the foreground task for any image (all *P>*.10; average *r*_arousal_  = .18; average *r*_time_  = .18). This analysis cannot unequivocally establish that attention was constantly focussed on the centre, especially since the box was always closed during the first 400 ms of image presentation. However, given that the fixation task was continuous and fast-paced, it is unlikely that differences in allocation of attentional resources during the experiment can account for differences in ratings or for predictive brain activity.

### Image rating task

Next, the rating data were analysed for all 24 images. The images showed substantial variability on both ratings dimensions. The correlations between *arousal* ratings and *time reference* ratings across participants for all images except one (image 12, *r* =  −.56; *P* = .02) were non-significant (all other *P*>.05, uncorrected; average correlation across all images: *r*_all_  = .04; *P*>.05; for all results see Table 1). We further correlated *arousal* and *time reference* ratings across all images for each participant separately. On average, the ratings were uncorrelated (average *r* = .08), and only two participants showed significant positive correlations between rating dimensions (participant 9: *r* = .43; participant 15: *r* = .64; both *P*<.05; all others *P*>.05). These results confirm that both dimensions were at least to a great extent independent of each other. Note that excluding these two participants from the multivariate regression analysis did not change any results.

### Low-level image features

Correlations were assessed between low-level features (colour, luminance, spatial frequency) of all 24 images and average rating scores across participants. No significant correlations were found for any dimension (for details see Table 2). Furthermore, none of the individual participants' ratings correlated with any of the image feature parameters (all Fishers-Z-transformed r<.20, *r*_crit_  = .55; see Table 2 for averaged results). Multiple regression analyses predicting *arousal* ratings (corrected *R*_cor^2^_  = .04, *F*(9, 14)  = 1.12, *P* = .41) and *time reference* ratings (*R*_cor^2^_  = .031, *F*(9, 14)  = 1.08, *P* = .43) were not significant. Note that the use of multivariate regression was precluded by the small number of images and features (N = 24) used here. Taken together, none of the results provided any evidence for a significant relation between ratings and any of the low-level visual features of the images.

**Table 2:** Correlation coefficients of Low-level Image Features and Ratings.

	Mean ratings				Individual ratings			
	Arousal		Time ref.		Arousal		Time ref.	
	*r*	*P*	*r*	*P*	Mean *r*_z_ *	SE	Mean *r*_z_ *	SE
Luminance								
Mean	.307	.157	−.022	.917	.156	0.041	−.011	0.039
SD	.001	.996	−.151	.484	−.001	0.047	−.109	0.036
Median	.322	.139	−.025	.909	.163	0.046	−.011	0.042
Colour								
R-value	.371	.089	−.146	.498	.190	0.046	−.092	0.037
G-value	.278	.200	.009	.967	.140	0.041	.008	0.040
B-value	.032	.881	.295	.174	.018	0.031	.195	0.040
R/G-ratio	.143	.509	−.425	.052	.083	0.053	−.272	0.049
R/B ratio	.273	.209	−.405	.063	.128	0.039	−.263	0.047
G/B ratio	.242	.263	−.315	.148	.112	0.038	−.206	0.046
Mean	.254	.242	.050	.818	.131	0.037	.037	0.038
Spatial frequency	.174	.420	−.026	.904	.070	0.057	−.033	0.045

Note: *r* =  Pearson correlation coefficients; *P* =  uncorrected significance level; SD  =  Standard Deviation of the Mean; ‘Mean ratings’ refers to tests between the average ratings (across all participants) and the low-level image feature parameters; ‘Individual ratings’ refers to the averaged correlation coefficients between the individual participants' ratings and the low-level image features (SE  =  Standard Error of the Mean; Mean *r*_z_  =  Fisher-Z-transformed *r*; *the critical value for *P*<.05, df  = 23) was *r*_crit_  = .553.

### CSD-ERPs

**Figure 2:**
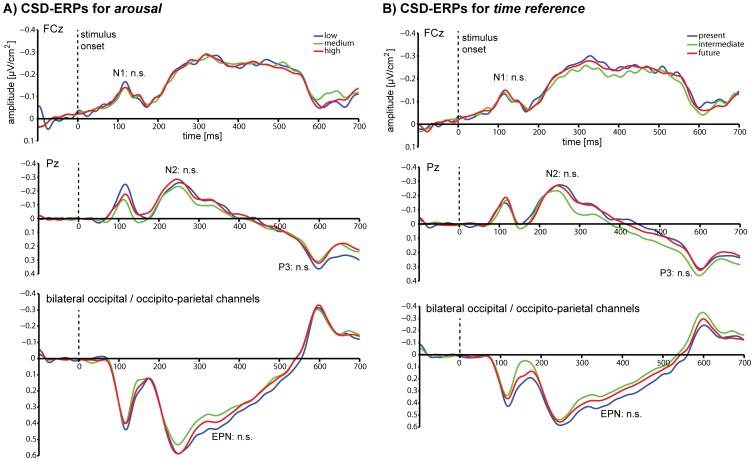
CSD-ERP results. Grand-average event-related potentials time-locked to stimulus onset, after current source density analysis (CSD-ERPs) sorted by rating increments for A) *Arousal* and B) *Time reference*. The classical CSD-ERP components were tested for significant differences between post-experimental rating increments at their typical sites: the CSD-N1 at FCz, the CSD-N2, the CSD-P3 at Pz, and the CSD-EPN as an average of P9, P10, PO7, PO8, O1, O2. None of the differences in amplitudes and latencies was significant for any comparison (see text for statistics).

The classical CSD-ERP components associated with stimulus processing were analysed at their typical channels where they were also found to be maximal (see Figure 2). These were the CSD-N1 at channel FCz, and the CSD-N2 and the CSD-P3 at channel Pz. For the CSD-EPN electrodes P9, PO7, O1, as well as P10, PO8, O2 were collapsed. For both dimensions, participants showed clear components for all rating increments. The distribution of number of trials for each rating increment used for the ERP analyses were as follows: arousal-high: mean  = 175.7; min  = 66, max  = 305; arousal-medium: mean  = 130.3; min  = 41, max  = 273; arousal-low: mean  = 70.1; min  = 16, max  = 168; time-present: mean  = 74.0; min  = 38, max  = 152; time-intermediate: mean  = 109.7; min  = 43, max  = 175; time-future: mean  = 192.5; min  = 80, max  = 295 (note that the SVR analyses were always performed using a balanced number of trials per rating increment condition).

#### Arousal

**Figure 3:**
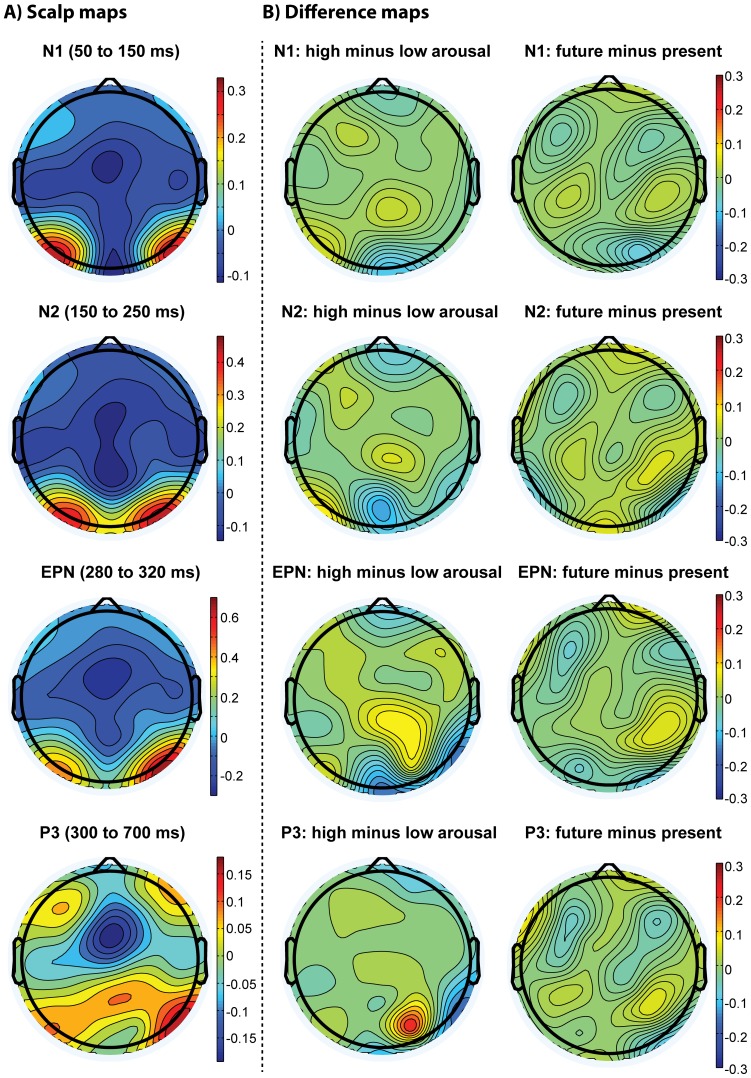
Scalp distributions of differences in CSD-ERPs. The left column displays scalp maps for the average distribution of voltage for the N1 (50 to 150 ms), the N2 (150–250 ms), the EPN (280 to 320 ms) and the P3 (300 to 700 ms) time windows. The middle column visualises the difference maps for *arousal* (high minus low), and the right column displays the difference maps for *time reference* (future minus present) for the same ERP components.

Repeated-measures ANOVA for peak amplitudes and latencies revealed no significant difference between rating increment conditions for the CSD-N1, the CSD-N2 and the CSD-P3 (for all details and statistics see Table 3 and Figure 3). For the CSD-EPN, repeated-measures ANOVA for mean amplitudes revealed no significant difference between rating increment conditions, and no condition-by-laterality interaction, but there was a significant effect of laterality (*F*(1, 14)  = 9.75, *P*<.01), indicating that EPN amplitudes were on average larger in the right than the left hemisphere (see Table 3).

**Table 3:** Means, standard errors of mean, and ANOVA results for the components of the event-related potentials for *arousal* ratings.

	Low		Medium		High				
	Mean	SE	Mean	SE	Mean	SE	*F*	*df*	*P*
*CSD N1 component*									
Peak Amplitude (µV/cm^2^)	−0.18	0.027	−0.17	0.021	−0.16	0.012	0.43	2, 28	.65
Latency (ms)	114.84	5.75	124.22	4.06	114.45	4.93	1.42	1.44, 20.19	.26
*CSD N2 component*									
Peak Amplitude (µV/cm^2^)	−0.31	0.043	−0.34	0.047	−0.30	0.043	0.91	1.38, 19.30	.39
Latency (ms)	214.45	9.29	225.00	7.05	222.40	8.60	0.75	1.36, 19.00	.44
*CSD P3 component*									
Peak Amplitude (µV/cm^2^)	0.45	0.094	0.35	0.047	0.37	0.047	0.84	1.06, 14.86	.38
Latency (ms)	603.00	5.75	603.13	5.77	603.47	7.11	0.30	2, 28	.75
*CSD EPN component*									
Left Hemisphere (µV/cm^2^)	0.35	0.070	0.32	0.056	0.34	0.054			
Right Hemisphere (µV/cm^2^)	0.50	0.083	0.51	0.068	0.49	0.071			
Rating increment							0.10	2, 28	.90
Laterality							9.75**	1, 14	<.01
Rating increment-by-laterality							0.63	1.38, 19.36	.54

Note: *df*s Greenhouse-Geisser corrected if required; SE  =  standard error of mean; **P<.01.

#### Time reference

A similar profile of results was found for the *time reference* dimension. Repeated-measures ANOVA likewise showed no significant differences between rating increment condition peak amplitudes as well as latencies for the CSD-N1, the CSD-N2 and the CSD-P3. The CSD-EPN repeated-measures ANOVA revealed no significant difference between conditions, and no condition-by-laterality interaction. There was a significant effect of laterality (*F*(1, 14)  = 9.19, *P*<.01), indicating that EPN amplitudes were on average larger in the right than the left hemisphere (see Table 4 and Figure 3 for all details and statistics).

**Table 4:** Means, standard errors of mean, and ANOVA results for the components of the event-related potentials for *time reference* ratings.

	Present		Intermediate		Future				
	Mean	SEM	Mean	SEM	Mean	SEM	*F*	*df*	*P*
*CSD N1 component*									
Peak Amplitudes (µV/cm^2^)	−0.16	0.020	−0.18	0.024	−0.17	0.017	0.46	2, 28	.64
Latency (ms)	119.66	6.35	115.76	4.66	177.71	2.59	0.22	2, 28	.80
*CSD N2 component*									
Peak Amplitude (µV/cm^2^)	−0.31	0.051	−0.27	0.048	−0.30	0.043	0.77	2, 28	.47
Latency (ms)	222.27	5.43	226.69	6.27	239.58	3.48	3.54	1.55, 21.73	.06
*CSD P3 component*	0.37	0.047	0.37	0.051	0.36	0.043	0.06	2, 28	.94
Peak Amplitude (µV/cm^2^)	611.07	6.98	607.68	7.65	606.77	7.08	0.30	1.18, 16.49	.63
Latency (ms)									
*CSD EPN component*									
Left Hemisphere (µV/cm^2^)	0.35	0.068	0.35	0.066	0.34	0.051			
Right Hemisphere (µV/cm^2^)	0.52	0.078	0.49	0.070	0.49	0.071			
Rating increment							0.25	1.46, 20.37	.71
Laterality							9.19**	1, 14	<.01
Rating increment-by-laterality							0.24	2, 28	.79

Note: *df*s Greenhouse-Geisser corrected if required; SEM  =  standard error of mean, **P<.01.

We further performed the same set of analyses on the same data without applying CSD analyses. The results were the same: we did not find any significant differences between any conditions for the N1, N2, P3 and the EPN, for either *arousal* or *time reference* (data not shown).

### Multivariate SVR

**Figure 4:**
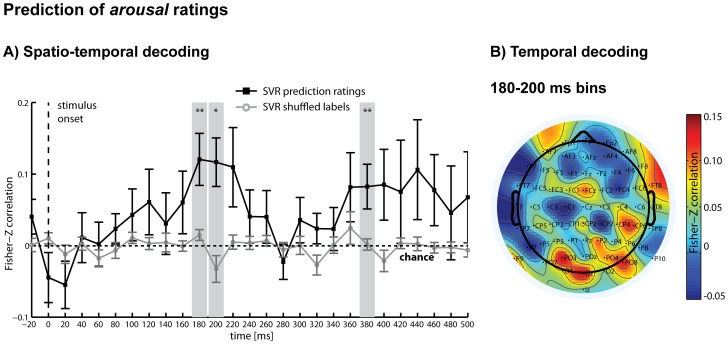
SVR results for arousal ratings. A) Spatio-temporal decoding: Multivariate support vector regression (SVR) was used to regress *arousal* ratings (in increments: low/medium/high) from distributed patterns of CSD-ERPs within analysis time windows of 40 ms that were moved through the first 500 ms of each trial in steps of 20 ms. Ratings could be regressed significantly above chance between 180–200 ms (denoting the central time points of the analysis windows) and again around 380 ms after stimulus presentation (black line). Additionally shown are the results of the same SVR analysis using shuffled labels to obtain an empirical chance distribution for statistical testing (grey line). B) Temporal decoding: Multivariate SVR was used for each channel separately to regress *arousal* ratings (in increments: low/medium/high) from purely temporal patterns of CSD-ERPs within the first significant time period of the spatio-temporal analysis (combined time bins 180 ms and 200 ms). The heat map illustrates predictive channels, with electrodes P9 (*t*(14) = 2.59*), PO3 (*t*(14) = 2.24*), O1 (*t*(14) = 2.46*), P6 (*t*(14) = 2.46*), PO4 (*t*(14) = 2.87*) reaching significance. *P<.05; **P<.01 (uncorrected); error bars  =  standard errors.

**Figure 5:**
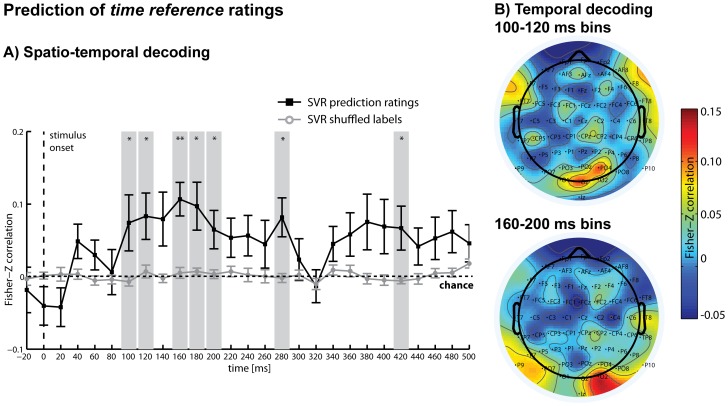
SVR results for time reference ratings. A) Spatio-temporal decoding: Multivariate support vector regression (SVR) was used to regress *time reference* ratings (in increments: present/intermediate/future) from distributed patterns of CSD-ERPs within analysis time windows of 40 ms that were moved through the first 500 ms of each trial in steps of 20 ms. Ratings could be regressed significantly above chance between 100–120 ms, 160–200 ms (denoting the central time points of the analysis windows) and again around 280 ms and 420 ms after stimulus presentation (black line). Additionally shown are the results of the same SVR analysis using shuffled labels to obtain an empirical chance distribution for statistical testing (grey line). B) Temporal decoding: SVR was used for each channel separately to regress *time reference* ratings (in increments: present/intermediate/future) from purely temporal patterns of CSD-ERPs within the first significant time periods of the spatio-temporal analysis (top panel: combined time bins 100 ms, 120ms; bottom panel: combined time bins 160 ms, 180 ms, 200 ms). Top panel: The heat map illustrates predictive channels, with electrodes FC5 (*t*(14) = 2.60*), CP5 (*t*(14) = 2.22*), Oz (*t*(14) = 2.63*), F4 (*t*(14) = 2.86*), O3 (*t*(14) = 3.97**) reaching significance. Bottom panel: Prediction reached significance for electrodes P9 (*t*(14) = 2.66*), PO7 (*t*(14) = 3.08**), CP2 (*t*(14) = 2.23*), PO4 (*t*(14) = 2.27*), O2 (*t*(14) = 3.46**); *P<.05, **P<.01 (uncorrected); error bars  =  standard errors.

While the deviations in single CSD-ERPs were not significantly different between rating increments at the channels, which showed the local maxima for these components, small differences in these and other channels, as reflected in the overall pattern of activity, were nonetheless predictive of the ratings. A linear SVR analysis showed that in the time period from 180 ms to 200 ms after stimulus presentation, the *arousal* ratings could be regressed significantly above chance, followed by a second period with highest accuracies at 380 ms (Figure 4A). The same analysis was then repeated for the post-experimental *time reference* ratings and showed that in the period from 100 ms to 120 ms and 160 ms to 200 ms after stimulus presentation, *time reference* rating increments could be predicted significantly above chance, followed by significant periods at 280 ms and 420 ms (Figure 5A). These results demonstrate that differences in the subjective judgements of *time reference*, independent of general differences in arousal triggered by the stimuli, were reflected in the distributed patterns of CSD-ERP waveforms, even though these differences were not detectable in selected single-channel grand-average CSD-ERPs. While both rating dimensions were mostly de-correlated on a behavioural level as demonstrated above, the rating information were decoded from the same early post-stimulus interval (approx., 100 to 400 ms after onset), pointing to a similar time period of processing.

These results were obtained by testing each analysis time-step against an empirical chance distribution of prediction results by repeating each SVR cross-validation and repetition step for each participant with randomly shuffled labels, providing a statistical test for each analysis time window separately. However, as single analysis time windows could not be regarded as independent (and might be based on the same underlying latent components), we also conducted additional, more general tests for the entire time period (first 400 ms), and for each half of this time period (20 to 200 ms, and 220 to 400 ms) by averaging across the respective prediction accuracies from each analysis time window (we acknowledge that these test do not speak to when information becomes available during the respective time periods, and that their definition is rather arbitrary). On average, analyses during the entire time period significantly predicted *arousal* ratings (*P* = .04), as well as *time reference* ratings (*P* = 0.008). *Arousal* ratings prediction was not significant when averaging across all tests in the first half (*P* = .1), but was significant for the second half (*P* = .02). *Time reference* prediction was significant after averaging for the first half (*P* = .02) as well as for the second half (*P* = .02) of the tests.

The results of the temporal decoding analysis are shown in Figure 4B for *arousal* and Figure 5B for *time reference*. For both *arousal* and *time reference*, high prediction accuracy was achieved mainly from occipital, occipito-parietal and parietal channels during these early significant time periods, while some fronto-central and frontal channels had moderate prediction accuracy. Note that this analysis did not take into account distributed information across channels that might have contributed to the spatio-temporal SVR results.

## Discussion

Our results showed that the stimulus dimension of *arousal* as well as the abstract dimension of *time reference* – the degree to which participants subjectively rated positive images to be related to the present or to the future after the experiment – could be predicted from brain activity recorded during passive visual stimulation. Differences in individual ratings were not strongly reflected in the classical early components of the CSD-ERP during the first several hundred milliseconds, which reflect different aspects of stimulus processing (e.g. [17]–[31]). Using a multivariate SVR analysis, however, ratings could be predicted from patterns of CSD-ERPs during this time period. Our results suggest that the initial perceptual stimulus analysis was accompanied (or quickly followed) by a fast, automatic semantic analysis that included *time reference*, without requiring the deliberate direction of attention towards this semantic dimension.

We found that ratings for both the *arousal* dimension and the *time reference* dimension could be regressed from distributed patterns of brain activity. *Arousal* was included as a control dimension, representing an aspect of the stimuli that can be accessed immediately and is strongly related to emotional and physiological responses [52], [53]. Emotional processing appears to be fast and automatic and takes place in the first few hundred milliseconds after stimulus presentation (e.g. [54], [55]). *Arousal* was therefore hypothesised to be likely to produce systematic differences in brain activity (e.g. [16], [17]). However, we did not observe strong arousal-related differences in single CSD-ERP components. The absence of strong ERP effects for *arousal* might have resulted from the distractor task, which could have prevented deeper emotional processing [55]. It could further be explained by the fact that, unlike other studies [17], ours did not include negative stimuli, leading to a restriction of possible emotional responses and a reduced spectrum of *arousal* ratings. Furthermore, we regressed individual *arousal* ratings for a rather small set of images as opposed to the norm ratings for a larger image set [17]. This means that after being exposed to our small stimulus set during the experiment, participants might have subjectively realigned their *arousal* ratings relative to the given twenty-four images. One might speculate that differences in *arousal* to other image stimuli not included in the present study might be more pronounced and, in turn, could potentially be associated with stronger differences in brain activity. Nonetheless, our more sensitive pattern regression analysis revealed that fine differences in *arousal* ratings were reflected in the overall patterns of ERPs during the early time period of visual processing.

We also showed that *time reference* ratings could be predicted from brain activity using multivariate pattern regression analysis. This dimension is more abstract than *arousal*, and was not expected to be associated with any specific emotional or physiological responses per se. In line with our findings, others have demonstrated that not all processing of stimulus dimensions requires deliberate attention to the respective features – or even to the stimuli in general – in order to be associated with brain activity. For example, it has been shown that post-experiment subjective ratings of attractiveness of faces was reflected in brain activity as measured with fMRI, even though during the experiment participants were only asked to judge their gender [11]. As in our study, participants were not aware of the post-experiment rating task later on. However, attractiveness ratings are likely to be based on pre-existing preferences [56] and might therefore result from the integration of other abstract semantic dimensions. In another study, brain activity reflected the processing of unattended images of scenes on a category level while participants attended to different images [12]. The categories of the unattended images in this study, however, were the same as were relevant for the attended images. Our study extends upon these results by demonstrating that brain activity can also reflect category information for unattended stimuli when participants are not aware of the category of interest. By making use of the excellent temporal resolution of EEG, we further showed that this information could be found within the first half-second after stimulus onset, pointing towards a fast and immediate process, rather than a subsequent re-evaluation. Note that we were not able to address whether this information would persist (or re-occur) during later stages of the stimulus exposure phase because the box opening started after four hundred milliseconds, which produced strong ERPs masking our signals of interest. The initial fast process that we observed might be driven by an automatic semantic analysis that extracts stimulus information that is likely to be important for everyday decisions, and might relate it to personal preferences. Such an early automatic evaluation mechanism would provide the decision-maker with a valuable shortcut for making fast decisions that involve rather complex choice options.

It might be argued that the dimensions *arousal* and *time reference* were confounded in our study. This explanation, however, is unlikely because the correlation analyses show that the two rating dimensions were mostly de-correlated on a behavioural level, and only two participants showed residual correlations. Another possibility is that only images with either reference to the present *or* to the future were perceived as positive, and our regression analysis predicted a valence-driven preference judgement rather than *time reference*. However, this is unlikely since all images were chosen such that they were invariably positive, i.e. they had a high positive valence score according to the norms [40]. In the present study, we deliberately aimed to exclude strong differences in valence-driven preference which might underlie differences in brain activity, a variable that might be related to predictive brain activity [13], [57]. In support of our argument that prediction of *time reference* was not related to differences in valence-driven preference, earlier studies found preference (as measured by interest ratings and viewing times) to be directly related to arousal [53], which, as argued above, was independent to *time reference* in our study. Our approach also more closely resembles delay discounting studies that focussed on positive, rewarding stimuli [4]–[8]. This leaves the possibility that other unknown factors related to the categories we chose might have confounded our regression results, which we cannot completely rule out. Notably, the IAPS stimuli [39] all depict semantically complex scenes or objects in colour and are not explicitly controlled for visual complexity or imbalance in other semantic categories. However, it is unlikely that visual complexity or colour perception were strongly associated specifically with *time reference*, as would be required to explain our findings. This is confirmed by our control analyses demonstrating that neither of the rating dimensions simply reflected low-level visual features of the images. Further arguments against this possibility are the variance in rating for individual images across participants and the absence of correlations between image features and ratings. Future studies should nonetheless assess a greater variety of rating dimensions to map out the space of subjective semantic judgements that can be predicted from stimuli with differing complexity under passive viewing conditions. We also did not explicitly control for eye position, which means that we cannot fully rule out that sometimes participants might have broken fixation and explored the background images. There is the possibility that this could have led to an uneven allocation of attention. This scenario, however, is not very likely given the demanding nature of the continuous foreground task. We also did not observe any strong or systematic eye movement artefacts in the EEG that would result from shifting gaze to the background images. It could also be argued that an uneven allocation of attentional resources to the background images could potentially also occur for peripheral vision. However, individual ratings were not significantly correlated with the accuracy in the foreground task for any image, speaking against this possibility. Most importantly, in any case, participants were unaware of the upcoming rating task and the images were completely irrelevant for the fixation task. This supports the conclusion that the processing of specific image dimensions took place without deliberate attention.

Showing that abstract stimulus dimensions can be predicted using multivariate techniques further demonstrates the validity of our method, which can detect subtle information in distributed patterns of ERPs that would otherwise be overlooked [32]. For example, differences in ERPs might not be reflected in strong and statistically significant differences in single electrodes (e.g. because of the involvement of deeper sources, or a multitude of brain regions) but in the distributed spatial and temporal pattern of ERPs across several channels. Note that classical ERP analysis, as we performed here, usually focuses on single channels that are known to clearly reflect interpretable ERP components [58]. While this remains the preferred approach for many research questions, multivariate analysis, on the other hand, is a more data-driven approach that facilitates the detection of systematic differences in activity patterns. As such, it is well-suited for exploratory investigations such as the present study. Furthermore, unlike earlier studies (e.g. [32], [33]), here we used a multivariate regression approach, which allowed us to establish a closer link between brain activity and the *degree* of subjective ratings, rather than just between different classes.

We conclude that our analysis was successful in predicting post-experimental *time reference* ratings from brain activity recorded during passive stimulus viewing, providing evidence that this specific stimulus dimension might be processed automatically, without deliberately directing attention. These findings are in line with reports that the ‘gist’ or general meaning of a scene can be extracted rapidly, as early as 70 ms after exposure to a stimulus [59], [60]. Moreover, we could predict directly from brain activity the extent to which individuals experienced some very abstract attributes of the stimuli that were not part of the explicitly depicted elements of the image (e.g. *time reference*). This automatic semantic analysis for the specific dimension of *time reference* might arise because of its importance for everyday decision-making. Specifically, many decisions that humans are confronted with involve choice options with outcomes that are available at different points in time. Thus, automatically extracting the relevance, or relation, of a stimulus to temporal aspects of reward might beneficially guide decision-making. Often, a positive stimulus itself might be a choice option (or, a reward), and its *time reference* could match or mismatch the current goal state of the decision-maker. However, sometimes there might simply be an overlap in semantic context (e.g. *time reference*) with other decision problems, for example financial decisions in which equally rewarding monetary choice options are available at different points in time [1], [2]. Being exposed to stimuli with a particular *time reference* could then activate the semantic concept of the present or the future, explaining why some stimuli can prime people to be more impulsive [4]–[8], or more patient [9], [10] in delay discounting tasks. Note that our study cannot decide whether the priming effects observed in other studies is due to the proposed mechanism as we speculate here, and the explicit link between our neural prediction findings and priming has to be investigated in future studies. However, our study provides evidence for the existence of neural signals that reflect the automatic extraction of *time reference* information from visual stimuli, thus making the explanation more likely.

Our results might also be relevant to explain findings from recent studies showing that even complex decisions, e.g. whether to buy a car or not, can be guided by cognitive evaluation processes which occur outside the decision-makers' awareness [61]. Such unconscious evaluation processes might be explained by an automatic processing of decision-related semantic information; these, in turn, would first require the automatic extraction of decision-related semantic information from relevant stimuli, as observed in our study. For example, it has been demonstrated that hypothetical purchase decisions for cars can be predicted from brain activity in medial prefrontal cortex and the anterior insula before participants were informed of having to make such a decision, during performance of a similar distractor task as used here [13]. Decision-relevant information about the cars could have been automatically extracted during passive exposure to the stimuli, as found in our study. Similarly, others asked their participants to make decisions about the quality of different apartments and prevented conscious deliberation using a demanding distractor task [62]. These authors have shown decision-related activation during the distractor task period in several brain regions, including visual cortices and lateral prefrontal cortex [62]. The preparation of such complex decisions would not only require an automatic re-processing of semantic decision-related information during a later decision phase, but most likely an early, automatic extraction of decision-relevant aspects from the stimuli during initial exposure, similar to what we observed for our positive images. Although our study did not address the transfer to decisions, it investigated its precondition and provided evidence that specific semantic stimulus information can indeed be rapidly and automatically extracted from the stimulus. This interpretation is further in line with recent findings demonstrating that abstract category information can be processed even when participants are not aware of the stimuli [63].

*Time reference* as an abstract stimulus dimension might be only one example of what can be processed automatically. Note that we preselected the stimuli with respect to whether they covered the present-future dimension in a pre-test. Indeed, many images (or scenarios in everyday life) might not be strongly related to any time period and therefore not trigger an automatic processing of this dimension. They might, however, be related to other core dimensions, which are relevant for typical everyday decisions that were not covered in our study. Future studies could further extend our findings by mapping out the semantic space that is reflected in brain activity during passive processing, and by investigating which brain regions code for semantic stimulus information. It would also be of interest to investigate whether the degree of information extractable from brain activity might be modulated by the context of the decision. Conversely, the degree of information about a specific stimulus dimension reflected in brain activity might also predict the automatic activation of decision goals [64], [65], or the formation of preferences when confronted with initially neutral stimuli [66].
